# History of Acute Promyelocytic Leukemia: A Tale of Endless Revolution

**DOI:** 10.4084/MJHID.2011.067

**Published:** 2011-12-21

**Authors:** Francesco Lo-Coco, Laura Cicconi

**Affiliations:** 1 Department of Biopathology, Tor Vergata University, Rome, Italy; 2 Laboratory of Neuro-Oncohematology, Santa Lucia Foundation, Rome, Italy

Only a few thousand people worldwide are diagnosed each year of Acute Promyelocytic Leukemia (APL). However, for a number of reasons, such rare disease is regarded as a paradigm in the entire field of medicine. Once considered the most malignant human leukemia as well as the one associated with the worst prognosis, APL has been transformed in the past few decades into the most frequently curable one. This extraordinary progress has been the result of an unprecedented coincidence of advances in both biological and clinical research. Today, APL represents a model for i) differentiation therapy, ii) tailored treatment targeting a molecular aberration, iii) value of minimal residual disease assessment as a surrogate of clinical outcome, and iv) translational research in general. In the following pages are reviewed the major discoveries and achievements in APL biology and therapy, with particular emphasis on those that have had more direct an impact in patient outcome. Finally, we briefly discuss some of the unsolved issues and future challenges in APL biology and treatment.

The first report on APL traces back to 1957, when the Norwegian hematologist LK Hillestad recognized APL as a distinct clinical entity, whose “*most outstanding feature was its very rapidly downhill course of few weeks’ duration, a white blood cell picture dominated by promyelocytes and severe bleeding caused mainly by fibrinolysis*”.^1^ A more detailed account of APL was given in 1959 by J Bernard (Hôpital St. Louis, Paris) who reported a series of 20 patients with the full definition of the disease and its association with promyelocytic proliferation, hyper-acute onset and catastrophic hemorrhagic events.^2^

Since the earlier reports, the life-threatening coagulopathy was recognized as the defining clinical feature of APL accounting for the majority of deaths at presentation and during initial cytotoxic treatment, with most fatal events being intracranial and pulmonary hemorrhages. The hemostatic abnormalities were attributed to a disseminated intravascular coagulopathy in which fibrinolysis and procoagulant activity triggered by APL blasts played a major role. As described by Rand *et al*, hematologic remission resulted in the resolution of the coagulopathy. 3 It is worth noting that, despite the impressive improvement in outcome achieved after the advent of *All*-trans retinoic acid (ATRA, see below) the early death rate in APL has remained elevated even in recent years, mostly because many patients die even before they can start treatment.^4^,^5^,^6^ This further highlights the importance of early recognition of the disease in order to promptly start anti-leukemic therapy and simultaneous transfusion support.

A seminal contribution in APL treatment was made in the early ‘70s, again by J Bernard and co-workers, who reported a dramatic raise in complete remission (CR) rates from 13% to 57% when using daunorubicin as monochemotherapy.^7^ Following this landmark study, anthracycline-based regimens were adopted in Italy, Spain and France during the ‘70s and ‘80s. APL was indeed recognized as a distinct entity in these countries long before the advent of ATRA and differentiation therapy, whereas in all other countries, including the USA, the disease was treated like the rest of AMLs until the early ‘90s, that is, without any specific distinction. Still nowadays, while novel experimental treatments are being tested and other agents have been shown to have high anti-leukemic activity in APL (e.g. arsenic trioxide, anti-CD33 antibodies), the standard recommended treatment for newly diagnosed APL consists of anthracycline monochemotherapy and ATRA.^8^,^9^ No other myeloid or lymphoid acute leukemia shows a similar pattern of chemosensitivity to a single agent, and, in fact, polychemotherapy schemes are the standard treatment in all non-APL acute leukemias. The biological reasons underlying such striking sensitivity of APL to anthracycline have not been clarified and should be a matter of investigation. Avvisati *et al*. reported in 1990 similarly high CR rates using idarubicin instead of daunorubicin.^10^

The French-American-British classification of 1976 clearly recognized APL (M3 type) and its peculiar morphologic features: these included marrow infiltration by dysplastic promyelocytes with heavy granulations and sometimes bundles of Auer rods ("faggots") accompanied by a strong myeloperoxidase (MPO) staining in the cytoplasm.^11^ Few years later, a microgranular variant (M3v) of the disease was identified and recognized in the updated FAB classification.^12^ This variant accounts for 20% of cases approximately. Subsequent biologic studies showed that compared to the more common hypergranular form, the M3v subtype carries identical molecular features, while phenotypically it is characterized by more frequent hyperleucocytosis and expression of CD2.^13^ Importantly, the variant form is equally responsive to anthracyclines, ATRA and arsenic trioxide (ATO), indicating that molecular identity corresponds to equal drug sensitivity in the two forms.

At the biological level, a major breakthrough in APL was the description by J Rowley from the University of Chicago of a reciprocal balanced translocation between chromosomes 15 and 17 t(15:17) (q21;q22).^14^ Subsequent studies showed that this translocation occurs in approximately 95% of APL cases and is unique to the disease. Karyotypic studies were pivotal to define APL as a genetic-clinical entity and paved the way for molecular characterization of the disease.

Despite the significant improvement in outcome obtained with anthracycline-based chemotherapy, cure rates were still between 25 and 50% up to the early ‘90s. An increase in CR rates was however achieved in the late ‘80s with the generalized adoption of intensive transfusion support (platelets and plasma) during induction therapy.

In the mid ‘80s a series of unrelated circumstances led to the first experiences with retinoic acid in China that brought about a revolutionary approach to cancer treatment. The inspiring inputs to initiate this novel strategy derived from i) *in vitro* studies on leukemic cell differentiation; ii) a philosophical lesson derived from Confucius who believed in the rehabilitation of criminals, and iii) a need of anticancer drugs less expensive than chemotherapy due to the economic constraints experienced at that time in China. The unusual combined results of experimental evidence, philosophical tradition and political-economic circumstances led to the first attempts in medicinal treatment to convert criminals (leukemic blasts) into good citizens (mature white blood cells) through the differentiating agent ATRA.

The first clinical experience with ATRA was reported in 1988 at the Shangai Rui-Jin Hospital under the guidance of ZY Wang. Twenty-four APL patients, of whom 8 previously treated and 16 newly diagnosed, were administered ATRA (45 to 100 mg/mq) as single agent therapy. Twenty-three patients achieved CR without exacerbation of coagulopathy and with no signs of myelosuppression.^15^ For the first time in anticancer therapy a dogma had been demolished–namely, the belief that a tumor always represents an irreversible condition. Notably, no information on the retinoic acid receptor being involved in the t(15:17) was available at that time. In other words, the use of ATRA preceded the cloning of retinoic acid receptor alpha and was therefore not driven by knowledge of the specific molecular defect affecting APL blasts.

The meeting that took place in 1985 in Paris between Shanghai’s scientists guided by ZY Wang and L Degos from the Hôpital St Louis allowed the beginning of a close collaboration between these two groups and facilitated the introduction of ATRA in Western countries. A student of Dr. ZY Wang’s, Z Chen, was carrying out his PhD at the Hôpital St Louis at the time. The striking results obtained in Shanghai were reproduced in France on a series of patients in first or successive relapse after chemotherapy, with CR rates of up to 63% and confirmed worldwide in larger cohorts of APL patients.^16^,^8^ Henceforth, Z Chen was involved, along with his wife Sai Juan, in biological and clinical studies of APL. They have since contributed to the scientific world a number of seminal studies on APL molecular pathogenesis, biologic characterization and treatment with ATRA and ATO. In 2003, Z Chen was appointed member of the USA National Academy of Sciences and since 2007 he has occupied the post of Chinese Minister of Health.

*In vitro* and *in vivo* studies carried out by R Warrell *et al* at Memorial Sloan-Kettering Hospital in New York showed that administration of ATRA at pharmacological concentrations (10^−6^ M) induced morphological maturation of leukemic cells, leading to complete remission. The differentiation process was characterized by the appearance of cells in intermediate stages of maturation expressing both immature and mature surface antigens (shown by flow cytometry) together with the t(15;17) as proven by sequential FISH analysis of primary cells from patients receiving ATRA.^17^

The molecular bases of ATRA response in APL were unraveled by genetic studies reported in the early ‘90s. The investigation results concomitantly published by three independent groups (coordinated by de H Thé, E Solomon and PG Pelicci, respectively) reported cloning of the t(15;17) translocation.^18^,^19^,^20^ The breakpoint on 17q21 was located within the locus of the Retinoic Acid Receptor alpha (*RARA*) gene. The partner gene located in the breakpoint region on chromosome 15q22, originally named *MYL*, was a previously unknown gene later on renamed Promyelocytic Leukemia gene or *PML*. This gene was subsequently shown to be involved in the control of cell proliferation, apoptosis and senescence.^21^ The availability of *in vitro* (NB4 cell line) and *in vivo* (transgenic mouse) models harboring the *PML-RARA* fusion gene fostered investigation on APL leukemogenesis. In particular, the work carried out by PP Pandolfi, PG Pelicci and RM Evans allowed for the unraveling of the biochemical mechanisms of transcriptional repression induced by the PML/RARA protein as well as the molecular basis of ATRA response.^22^,^23^,^24^ Meanwhile, the cloning of the t(15;17) also enabled the design of PCR-based strategies to be used for rapid diagnosis as well as for sensitive detection of minimal residual disease.^25^,^26^,^27^

At the clinical level, it soon became clear that the outstanding results obtained with ATRA in terms of CR rates were not paralleled by satisfactory outcomes in the long-term. This led to the design of ATRA plus chemotherapy trials that were conducted in Europe, the USA and Japan by multicenter cooperative groups. The results of these studies, which were recently updated on a very long-term follow-up, indicate that the simultaneous use of ATRA and anthracycline-containing chemotherapy is the best option as it can cure up to 75%–80% of newly diagnosed patients.^8^,^9^ A consensus based on these studies established simultaneous administration of ATRA plus chemotherapy as the gold standard in frontline therapy of APL.

Meanwhile, the amplification of PML-RARA fusion transcript by RT-PCR approach provided high sensitivity and specificity for rapid and reliable diagnostic results and enabled the detection of the precise type of breakpoint on the PML gene so as to better assess minimal residual disease (MRD). The clinical importance of PCR-based diagnosis and MRD monitoring in APL was initially suggested by studies carried out in the US and in Italy.^25^,^26^ These showed that persistence or reappearance of PCR positivity for PML/RARA in patients with morphological remission was correlated to impending hematological relapse.^25^ Subsequent observations on the same line were reported in England by D Grimwade and by several other investigators worldwide.^27^,^28^ The body of information on outcome correlation derived from longitudinal PCR studies in the context of clinical trials offered the possibility to identify patients requiring treatment intensification and to adopt “pre-emptive” therapeutic strategies in Italy and the UK.^29^,^30^ Notably, this approach proved useful in avoiding early hemorrhagic deaths at time of relapse and improved overall outcome. The recent adoption of modern real-time quantitative PCR for PML/RARA detection allowed for better investigation of the kinetics of response and disease relapse as well as establishing a rationale for sample collection timing, as shown in a large study from the MRC reported by Grimwade *et al*.^30^

The trials conducted in the 1990–2000 decade provided an important source for the investigation of prognostic factors to be used for treatment stratification. In particular, the so-called Sanz’s score was developed to dissect relapse risks for patients receiving AIDA-like regimens adopted by the GIMEMA and PETHEMA.^31^ This in turn allowed the design of distinct strategies which were aimed at sparing unnecessary toxicity for patients with WBC counts inferior to 10×10^9^/L, whereas more intensive post-induction chemotherapy including cytarabine were adopted for high risk (hyperleucocytic) patients. The results of both GIMEMA and PETHEMA studies using a risk-adapted approach showed significant improvement of outcome.^32^,^33^ Interestingly enough, ever since the early times of APL history, J Bernard identified fibrinogen levels and WBC count as two important prognostic factors in APL.^34^

Other large trials conducted by the French-European APL Group, the British MRC (now NCRI), the Japanese JALSG and the German AMLCG confirmed the advantage of risk-adapted strategies using mainly WBC count as a prognostic factor. Overall, studies reported in the last few years with ATRA and risk-adapted chemotherapy resulted in CR rates of up to 95% and OS rates >85%.^8^,^9^,^35^

In summary, a number of significant advances contributed to achieve the extraordinary cure rates in APL mentioned above, converting this once rapidly fatal leukemia into the most curable one. The advances can be chronologically summarized as such: i) Advent of ATRA and its inclusion in front-line combinatorial treatments together with anthracycline-based chemotherapy; ii) Improvements in supportive care; iii) Diagnostic molecular refinement and sensitive MRD monitoring by PCR; iv) Definition of relapse-risk categories and adoption of risk-adapted strategies, and v) Pre-emptive therapy of disease relapse.

In spite of such impressive progress, new revolutionary facts were yet to come.

In 1994, J Bernard described the history of APL as a series of battles to combat dogmas. This extraordinary man indeed promoted, anticipated and witnessed many revolutionary achievements in APL’s history. Despite his long life, he died shortly before one last dogma was about to be demolished. The recent advent of an old revisited remedy, arsenic, as the most effective single agent in APL, brought up the potential to cure leukemia without chemotherapy.

Arsenic compounds had been used as therapeutic agents for more than 2,000 years in Western and Eastern medicine, particularly in China. The efficacy of arsenic as an anti-leukemic agent was initially demonstrated in Boston in 1880s when an arsenic solution was used to reduce leucocytosis. Half a century later, arsenic proved to be effective in Chronic Myeloid Leukemia and was widely used to treat this condition before the introduction of standard chemotherapy. Despite the advent of novel cytotoxic drugs, the empirical use of arsenic as an anti-leukemic agent continued in China throughout the past century.

Initial results on APL treatment with ATO were published in the late ’90s by Chinese investigators who observed striking responsiveness only in this subset among several leukemia types assessed. In particular, a study from Harbin Medical University reported CR rates with ATO as a single agent of up to 73% and 50% in newly diagnosed and relapsed APL patients respectively. Similar results were published by investigators from Shanghai in a series of APL patients in relapse after first-line treatment with ATRA.^36^,^37^

These preliminary results encouraged the design of a multicenter trial in the US in which ATO was administered as salvage therapy in relapsed patients. Eighty-five percent of the 40 patients treated with single agent ATO achieved hematological CR and 86% of the assessable patients achieved molecular complete remission after consolidation cycles.^38^ A number of subsequent studies conducted in Europe, India and Iran confirmed these observations and encouraged the use of ATO as first line therapy in light of its efficacy coupled with a mild toxicity profile.^39^

Meanwhile, several groups investigated the molecular mechanism underlying the efficacy of ATO in APL. These studies showed a dual mechanism of action inducing partial differentiation at low concentrations and a pro-apoptotic effect at higher doses. As demonstrated by Chen *et al* and Shao *et al*, at low concentrations, ATO exerts a partial differentiating effect by inducing the sumoylation of both PML-RARA and PML, leading to their degradation through the proteasome pathway. On the contrary, at high concentrations ATO induces apoptosis through caspase activation, ROS production and induction of the mithocondria-mediated intrinsic apoptotic pathway.^40^,^41^

Shen *et al* and Hu *et al* reported that, compared to ATRA alone, the ATO plus ATRA combination resulted in earlier CR achievement and platelet recovery, more rapid reduction of the PML-RARA transcript and lower relapse rates in newly diagnosed APL patients.^42^,^43^ Similar results were reported by Estey *et al* from the MD Anderson Cancer Center in Houston.^44^ Finally, a recently terminated large randomized trial conducted in the USA, showed a benefit from adding ATO to standard ATRA and chemotherapy during consolidation in front-line therapy.^45^

The suggested synergism of the ATRA plus ATO combination was exploited in the design of two prospective randomized studies in newly diagnosed patients that are currently being carried out by the Italian-German groups GIMEMA-SAL-ALSG (APL0406 study) as well as by the British NCRI. In both cases, a totally chemotherapy-free approach (experimental arm) is being tested against the standard AIDA-like regimen. In particular, the Italian-German study investigates in the experimental arm the efficacy of the prolonged ATO+ATRA scheme developed by E Estey *et al*^44^ together with longitudinal PCR-monitoring as a safety criterion to adopt pre-emptive salvage for patients who undergo molecular relapse. It is expected that the results of these randomized studies will better clarify whether APL can be cured without chemotherapy.

Although, after half a century of revolutionary events in biological and clinical research APL has become the most curable of acute leukemias, many challenges remain for investigation and several issues are still unsolved. In addition, both in developing and developed parts of the world many patients still die before treatment or during the first days after the initiation of therapy. Improvements in early death rate require a number of efforts in education on disease characteristics, epidemiologic studies using population registries as well as investigation on the coagulopathy and predictive factors of major hemorrhagic events.

Despite the promise of differentiation therapy, more than 20 years after the advent of ATRA we have not been able to extend this approach beyond APL and no differentiation therapy has been successfully applied to other leukemia subsets or solid tumors. This underscores our limited understanding of the molecular mechanisms underlying leukemic cell differentiation in response to retinoids. Similarly, the mechanism of action of ATO is not fully deciphered.

In conclusion, as a model disease that has continuously inspired investigation generating important insights, it can be said that APL’s future history may still have novel unexpected discoveries in store and so add new thrilling chapters to this revolutionary tale.
